# Eurekometrics: Analyzing the Nature of Discovery

**DOI:** 10.1371/journal.pcbi.1002072

**Published:** 2011-06-30

**Authors:** Samuel Arbesman, Nicholas A. Christakis

**Affiliations:** 1 Department of Health Care Policy, Harvard Medical School, Boston, Massachusetts, United States of America; 2 Institute for Quantitative Social Science, Harvard University, Cambridge, Massachusetts, United States of America; 3 Department of Sociology, Harvard University, Cambridge, Massachusetts, United States of America; 4 Department of Medicine, Harvard Medical School, Boston, Massachusetts, United States of America; University of California San Diego, United States of America

Until recently, the quantitative study of science has focused on studying patterns in publications [1], [2], such as citation counts to discern impact, and in coauthorship networks to discern collaboration. However, two major trends are converging that offer the field of scientometrics a novel opportunity to understand scientific discovery and also to influence how science is done. The first is the advent of vast computational resources and storage capacity available to scientists [3], [4], and the second is automated science [5], [6]. These innovations offer the potential for a new type of scientometrics: quantitatively examining scientific *discoveries* themselves. This study of discoveries, rather than simply of scientific publications, offers the opportunity to understand science at a deeper level. We term this discovery-based approach to scientometrics as *eurekometrics*.

Eurekometrics aims to supplement the traditional bibliometric approach of scientometrics by examining the properties of scientific discoveries themselves rather than examining the properties of scientific publications. This is not simply a methodological development but a conceptual one. By using new types of data, we may be able to ask entirely different sorts of questions than we could before. For example, we are now able to examine both the material properties of phenomena that are discovered, such as their physical size, intrinsic entropy, or informational complexity, as well as the human properties of the phenomena, such as how much money, time, or effort it takes to discover them.

For instance, a traditional scientometric approach to understanding the nature of the genetic code and its elucidation would be to study the publications relevant to this area, looking at the citation network among these papers, for example. However, a eurekometric approach would instead examine the properties of the discoveries that were made during the deciphering of the code. In the 1960s, there was a large-scale push to elucidate what each triplet codon sequence coded for [7]. Using a simple metric for informational entropy [8], one can examine the properties of each codon and find out whether or not, on average, the coding of those codons with less entropy can be found using more types of experiments [7]. In other words, a simple eurekometric approach could examine whether or not those codons with less information can be more easily understood.

**Figure 1:**
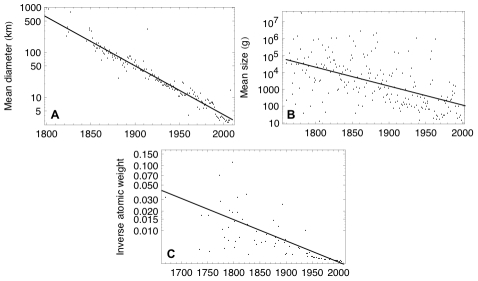
Ease of scientific discovery over time. (A) Mean diameter (kilometers) of minor planets discovered, 1802–2008. (B) Mean physical size (g) of mammalian species discovered, 1760–2003. (C) Mean inverse of atomic weight of chemical elements discovered, 1669–2006. Adapted from [9].

There are already examples of eurekometrics beyond the foregoing one. Using the properties and dates of discovery of mammalian species, minor planets, and chemical elements, a quantitative measurement of the decay in ease of scientific discovery has been made [9] (see Figure 1). By using measurements of the size of each item, a crude proxy for difficulty of discovery was developed. This allowed for insight into whether discovery becomes easier with time, and an analysis of how discoveries actually proceed over time. In addition, examination of the properties of scientific discoveries can be used to predict future discovery. For example, by examining the properties of previously discovered extrasolar planets, a prediction for the first potentially habitable planet similar to Earth has been made [10]. A video visually displaying the location of minor planet discoveries from 1980 to 2010 relative to the Earth's orbit also offers eurekometric insight [11].

Furthermore, there are examples of research that has begun to bridge the gap between bibliometrics and eurekometrics. Using gene interaction data from high-throughput experiments combined with citation data, an attempt was made to understand the relationship between the reliability of reported interactions and the popularity of a research field [12]. These researchers also examined how the importance of a gene in interaction networks is related to its popularity in the literature [13].

With the increase of automated discovery and large-scale data collection, eurekometric research has the potential to explode. First, automated science will necessarily have the property of creating large amounts of discovery data. Illustrative examples of automated science include the Sloan Digital Sky Survey [14], Lincoln Near-Earth Asteroid Program [15], Gordon and Betty Moore Foundation Marine Microbial Genome Sequencing Project [16], and the Census of Marine Life [17]. The initial output of these projects will not be publications, but findings. Each object, such as a newly discovered asteroid, need not have its own publication, but each object can be examined separately from a eurekometric perspective.

In addition, there is the potential in such areas as automated drug discovery [18], automated chemical synthesis path discovery [19], and automated theorem proving [20]. In all these cases, the conceptually informed and rigorously quantifiable analysis of what is discovered, and when, will shed light on many things, e.g., where there is a relationship between the object of inquiry and human effort.

In addition, other types of research projects will provide potential for eurekometrics. For example, citizen science research, where interested laypeople provide much of the scientific labor, also has potential. Such projects include Galaxy Zoo [21], which examines stellar phenomena; Foldit [22], which studies protein folding; the Audobon Christmas Bird Count [23], which catalogues birds; and Valley of the Khans [24], which hunts for Genghis Khan's tomb. In addition to providing vast amounts of discovery data, these projects will allow us to understand the way collaborative approaches can create further discovery and the properties of discoveries that are best suited to citizen science.

Despite the great strides in automated discovery and digitization of data that is currently occurring, however, there are limits to eurekometrics. The most important limitation is how to determine what constitutes a “discovery.” Quantifying what constitutes a discovery is never an easy proposition: Is each publication a discovery? Or do only certain ones rise to meet that definition? Furthermore, even if we can list discoveries, it needn't necessarily be possible to quantify their properties. For example, while it's possible to quantify the properties of minor planets and extrasolar planets, it is not nearly as easy to quantify the properties of methodological innovations made in computational fields.

Scientometrics has for too long focused on understanding scientific progress at the level of the publication. Eurekometrics will allow us to understand the pace and determinants of scientific discovery in a way that simply examining the patterns in publications will not. For the first time, we will be able to explore how the properties of nature yield to human science.
